# Interpretable machine learning for identifying co-occurring internet addiction and maladaptive cognitive emotion regulation in older adults: a cross-sectional study

**DOI:** 10.3389/fpubh.2026.1901765

**Published:** 2026-08-13

**Authors:** Jingrui Gui, Haiyue Chen, Jiazhao Li, Junrong Wang, Yihan Wang, Ruoxi Zhang, Xinya Zhang, Huashan Chen, Xu Zhang, Jing Zhang, Wenjuan Wang

**Affiliations:** Bengbu Medical University, Bengbu, Anhui, China

**Keywords:** internet addiction, machine learning, maladaptive cognitive emotion regulation strategies, older adults, SHAP

## Abstract

**Background:**

Internet addiction and maladaptive cognitive emotion regulation may co-occur in later life, yet evidence-based approaches for identifying this co-occurrence remain limited. This study developed and interpreted a machine learning classifier to distinguish community-dwelling older adults with versus without current co-occurrence. A cross-sectional survey was conducted among 1,471 adults aged 60 years or older in China. Candidate predictors included demographic characteristics, general well-being, family functioning, and social support.

**Methods:**

After preprocessing, correlation-based collinearity screening and ten-fold cross-validated LASSO regression were used for feature selection. Five models, including logistic regression, random forest, support vector machine, gradient boosting machine, and multilayer perceptron, were developed using the training set and internally validated in a randomly held-out test set. Model performance was evaluated using discrimination, calibration, and clinical utility metrics, and SHAP was applied for model interpretation.

**Results:**

Eight predictors were retained: general well-being, support utilization, age, gender, educational attainment, marital status, objective support, and living with children. The gradient boosting machine (GBM) showed the best overall performance in the test set, with an AUROC of 0.904, accuracy of 0.816, sensitivity of 0.810, specificity of 0.818, and Brier score of 0.118. SHAP analysis identified general well-being as the dominant predictor, followed by educational attainment and objective support. Lower well-being, older age, male gender, higher educational attainment, and not being married or partnered were generally associated with a higher model-predicted probability of current co-occurrence; higher support utilization was also associated with a higher predicted probability, particularly at lower levels of general well-being.

**Conclusion:**

This interpretable classifier may facilitate the identification of co-occurring conditions among community-dwelling older adults. Given its cross-sectional design, the model distinguishes current co-occurrence status rather than predicting future onset.

## 1 Introduction

Against the backdrop of an intensifying aging process and the ubiquitous adoption of digital technologies in China, the convergence of digitalization and population graying has emerged as a salient global trend. In recent years, internet use among older adults has increased substantially, with digital technology becoming deeply embedded in daily life as a primary medium for information seeking, leisure, and social interaction ((1)). Researchers commonly conceptualize internet addiction as a form of problematic or poorly controlled internet use associated with functional impairment. According to the Uses and Gratifications Theory (UGT), older adults may actively use digital media to compensate for unmet offline needs (2, 3). Older adults frequently encounter loneliness, shrinking social networks, and health-related anxiety in offline contexts. When these psychosocial needs remain unmet, compensatory internet use may emerge, potentially escalating into addictive behaviors.

Satisfying the needs for autonomy, competence, and relatedness drives psychological growth according to Self-Determination Theory (SDT) (4). In later life, diminished social role engagement and declining functional capacity may weaken self-worth. Although the accessibility and interactivity of the internet can partially compensate for unmet psychosocial needs, prolonged reliance may progressively escalate into internet addiction. Declines in subjective cognitive capacity may further contribute to memory problems, social withdrawal, and perceived neglect, thereby intensifying isolation. When confronted with multiple stressors, such as aging, illness, and role transitions, older adults adopt different coping strategies, and previous research has identified passive coping as an important predictor of addictive behaviors. Consistent with Stress-Coping Theory, online immersion may help alleviate negative emotions triggered by daily stress (5). When older adults lack effective real-world coping resources, they may turn to the internet as a maladaptive emotional buffer to escape negative affect. Although immersion in online activities may temporarily relieve anxiety and loneliness, sustained reliance may heighten vulnerability to internet addiction.

In the progression from real-life psychological distress to internet addiction, emotion regulation difficulties have emerged as a critical mediating factor (6). Maladaptive cognitive emotion regulation strategies constitute a key subdomain of emotion regulation research, referring to cognitive regulatory patterns that are inconsistent with situational demands, fail to alleviate negative affect effectively, and may even exacerbate psychological distress in response to adverse life events or negative emotions. Emotion regulation difficulties have been consistently associated with mental health problems in adulthood (7). According to the Process Model of Emotion Regulation (PMER), maladaptive cognitive emotion regulation may reflect inappropriate strategy deployment during the cognitive change and response modulation stages (7). Rather than alleviating negative emotions, maladaptive cognitive emotion regulation intensifies psychological distress and precipitates subsequent maladaptive behaviors.

The cognitive emotion regulation framework further distinguishes adaptive from maladaptive regulatory strategies and emphasizes that individual appraisals shape emotional responses (8). When individuals rely rigidly or excessively on cognitive responses to negative events without sufficient contextual flexibility, chronic use of maladaptive strategies during late-life stressors, such as declining health, role loss, and social isolation, may foster negative cognitive biases, erode psychological resilience, and increase reliance on avoidant coping. This process may, in turn, heighten vulnerability to problematic behaviors, including internet addiction. The transition to later life involves role changes and health decline, both of which substantially increase demands for emotion regulation. Declining cognitive resources and reduced social support may further predispose older adults to maladaptive strategies for managing stress and negative affect. By providing emotional release, immediate gratification, and virtual escape, the internet may serve as a maladaptive outlet for emotion regulation, enabling older adults to avoid real-life stressors. Chronic engagement in such online avoidance therefore carries significant risk for addiction (9), potentially establishing a self-perpetuating cycle linking negative emotions, maladaptive regulation, and internet addiction.

As healthcare data become increasingly complex, traditional statistical methods may have limitations when developing classification models for complex psychological outcomes. Conventional approaches, such as LR, are primarily suited to modeling simple linear associations and are less effective at capturing complex nonlinear interactions among variables. They also often rely on subjective judgment in variable selection, which may compromise model performance and generalizability (10). Structural equation modeling is highly effective for theory testing; however, it remains inadequate for capturing the complex nonlinearities and multivariable interactions frequently observed in actual data. The evolution of machine learning techniques has paved the way for overcoming these methodological hurdles. Machine learning serves as a robust analytical framework for extracting complex structures from diverse, high-dimensional data independently of pre-established heuristic rules. Its main advantages include robust nonlinear modeling, improved generalization through cross-validation and regularization, and the capacity to handle large-scale data with automated feature engineering (11).

Machine learning is now widely used in risk prediction research in mental health and psychology (12). Regarding geriatric mental health, researchers used the China Health and Retirement Longitudinal Study (CHARLS) database to develop depression risk prediction models with eight machine learning algorithms. They found that eXtreme Gradient Boosting (XGBoost) achieved the best performance, with a mean AUROC of 0.69 (13). Another study using the NHANES database to examine depression screening in older adults compared seven machine learning algorithms and likewise identified XGBoost as the best-performing model, with an accuracy of 0.82 and an AUC of 0.88 (14). In the field of addictive behaviors, emerging studies have also applied machine learning to identify risk factors for excessive smartphone dependence, with XGBoost showing superior predictive performance among older adults (accuracy = 0.925) (15). Beyond model development, recent longitudinal evidence showed that positive exercise experiences predicted lower subsequent mobile phone addiction tendencies among older adults, highlighting the relevance of exercise-related experiences to later-life behavioral addiction (16). A person-centered study further identified heterogeneous alexithymia profiles among older adults, with these profiles differing in physical activity and maladaptive eating behaviors, underscoring psychological heterogeneity in later-life health behaviors (17). Recent aging-cohort studies have demonstrated that prospective prediction requires temporally ordered measurements and follow-up outcomes, whereas broader generalizability can be evaluated through independent or cross-cultural cohort validation (18–20). The present dataset was originally collected as a single-wave community survey without follow-up outcomes. Accordingly, rather than estimating incident future risk, this study focused on the cross-sectional identification of current co-occurrence status. Commonly used machine learning algorithms include logistic regression (LR), random forest (RF), support vector machine (SVM), gradient boosting machine (GBM), multilayer perceptron (MLP), LightGBM and XGBoost (21). In particular, ensemble learning algorithms such as XGBoost and RF often achieve superior predictive accuracy by combining multiple weak learners (21, 22).

Although existing research has begun to examine internet use among older adults, most studies have focused on smartphone overdependence or general internet addiction. Researchers have paid relatively little attention to the underlying mechanisms linking internet addiction in later life to its characteristic psychological features, particularly maladaptive cognitive emotion regulation strategies. As a key psychological factor in the context of aging, loneliness, and health anxiety, maladaptive cognitive emotion regulation offers a critical lens for understanding the development and maintenance of internet addiction in later life. Yet, researchers have rarely incorporated it as a core feature in machine-learning classification models. In addition, existing research is limited in two important respects: firstly, most studies have focused on addictive behaviors or emotion regulation in isolation, with little integrated analysis from the perspective of co-occurrence. Second, traditional statistical methods may be insufficient for capturing nonlinear interactions among multidimensional psychological variables, and limited model interpretability may further hinder clinical translation.

Recently, SHAP, a method grounded in game theory, has emerged as a powerful approach for addressing these limitations. SHAP not only evaluates model outputs but also quantifies the direction and magnitude of individual feature contributions, thereby improving the transparency of model interpretation and rendering “black box” models more clinically understandable. By identifying the features contributing to the model-predicted probability of current co-occurrence, this approach may improve the transparency and interpretability of the classification model.

Building on these considerations, this study integrates survey data with multiple theoretical perspectives. The Process Model of Emotion Regulation serves as the central framework, while SDT and Stress-Coping Theory provide complementary perspectives on internet-use motivation and coping behavior. Given the cross-sectional design, this study did not aim to predict future onset. Instead, we systematically benchmarked several machine-learning models to identify current co-occurring internet addiction and maladaptive cognitive emotion regulation among older adults. SHAP analysis was further employed to identify influential model features and provide global and individual-level explanations of the classification results.

## 2 Methods

### 2.1 Study participants

**Figure 1:**
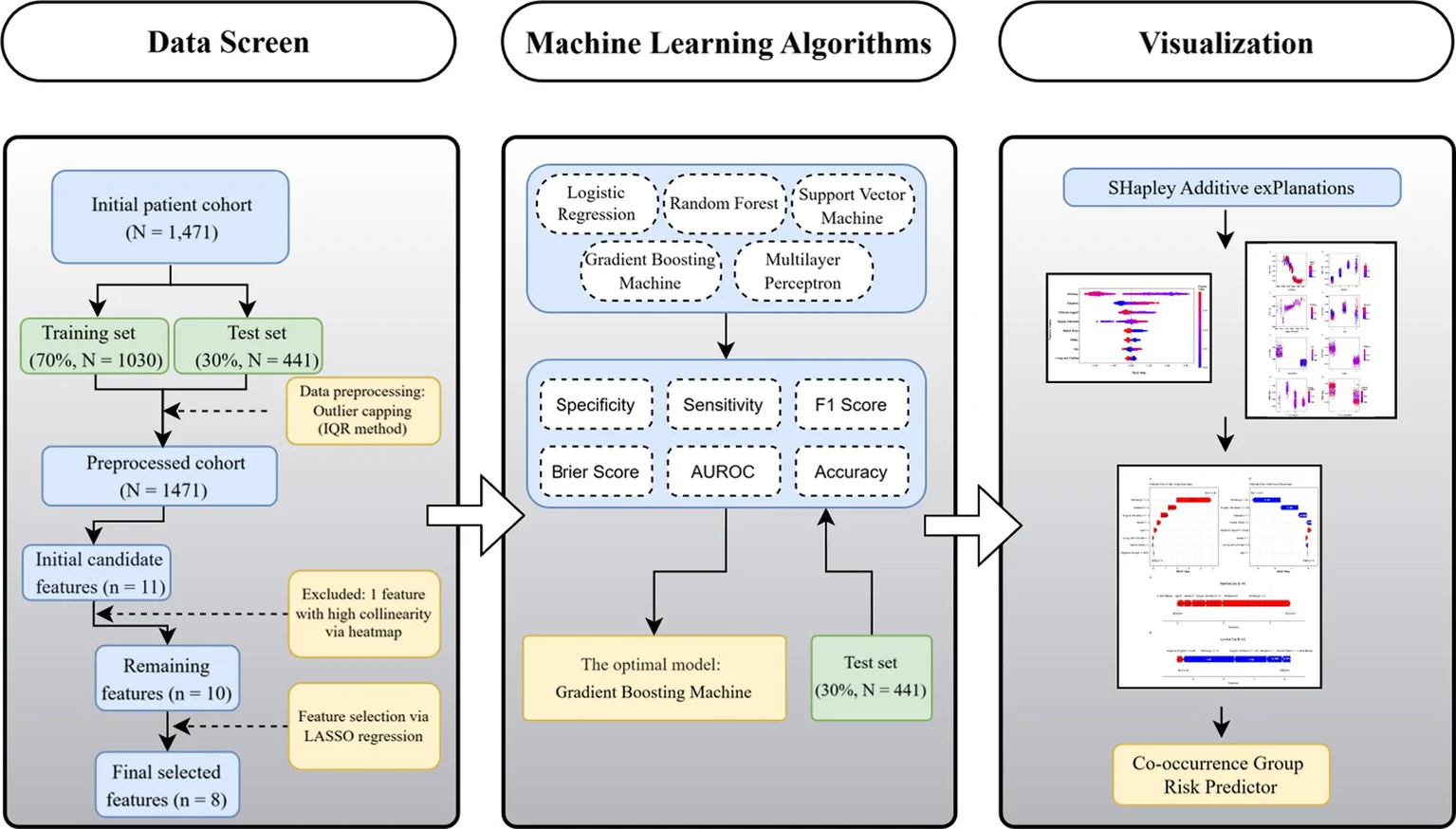
Flowchart of the study. The flowchart summarizes the overall analytical workflow of the study, including data preprocessing, feature selection, machine learning model development, performance evaluation, and SHAP-based interpretability analysis with visualization. First, the original cohort was randomly partitioned into training and testing subsets using a 70/30 split, followed by outlier processing and standardization calculated strictly on the training subset. A two-step feature selection strategy, comprising correlation analysis and LASSO regression, identified the core predictors. Based on these features, we developed and compared five machine learning models: LR, RF, SVM, GBM, and MLP. We evaluated model performance using AUROC, accuracy, sensitivity, specificity, F1 score, and the Brier score. Finally, the SHAP framework enabled both global and local interpretation of the optimal model, supporting the deployment of a web-based classification interface for individual-level assessment of current co-occurrence.

Adopting the age threshold set by the World Health Organization Regional Office for the Western Pacific (2021), this study defines “older adults” as individuals aged 60 years or above. We conducted a cross-sectional survey using cluster random sampling among community-dwelling older adults in Anhui Province, China, between July and September 2025. All candidate features and the co-occurrence outcome were assessed within the same survey wave; therefore, the analysis was designed to classify current co-occurrence status rather than predict future onset. A total of 1,471 valid questionnaires were collected. The sample included 673 men (45.75%) and 798 women (54.25%). In terms of age distribution, 830 participants (56.42%) were aged 60–70 years, 431 (29.30%) were 71–80 years old, and 210 (14.28%) were aged 81 years or above. Regarding living arrangements, 770 participants (52.35%) lived with their children, whereas 701 (47.65%) did not. Educational attainment varied: 574 participants (39.02%) had completed primary school, 428 (29.10%) junior high school, 290 (19.71%) senior high school, and 179 (12.17%) college education or above. In addition, 998 participants (67.85%) reported having a spouse or partner, whereas 473 (32.15%) did not. Before enrollment, we informed all participants of the study’s purpose, procedures, privacy protection policies, and the option to discontinue participation at any stage. The Ethics Committee of Bengbu Medical University approved the study protocol in 2024 (approval number: [2024] No. 411), and we conducted data collection in 2025 in accordance with the Declaration of Helsinki. To establish a rigorous, leak-free validation workflow, the raw dataset (*N* = 1,471) was initially partitioned into a training cohort (70.0%, *n* = 1,030) and a held-out test cohort (30.0%, *n* = 441) in a 7:3 ratio prior to any downstream data preprocessing or feature transformation (see Figure 1).

### 2.2 Study variables

#### 2.2.1 Outcome variables

We assessed internet addiction using the 17-item Diagnostic Scale for Internet Addiction Disorder (DSFIAD), compiled by Liu et al. (23), and applied a total score cutoff of 45 to determine positive internet addiction status among older adults. This native instrument captures two core dimensions: internet addiction symptoms (13 items screening features such as withdrawal and tolerance) and internet addiction triggers (4 items identifying psychological risk factors). Responses are scored on a 5-point Likert scale from 1 (no) to 5 (always). The Cronbach’s alpha for the DSFIAD in this sample was 0.954.

We measured maladaptive cognitive emotion regulation strategies using the Chinese adaptation of the CERQ, originally developed in the initial CERQ study (24) and subsequently validated in China (25). Although the comprehensive 36-item tool encompasses nine distinct cognitive domains, this analysis focused explicitly on the maladaptive component, which is evaluated across four specific subscales: self-blame, rumination, catastrophizing, and blaming others. Higher subscale scores indicate a stronger propensity to deploy these negative cognitive strategies. The Cronbach’s alpha coefficients reached 0.919 for the complete inventory and 0.885 for the maladaptive dimension. We defined co-occurrence as simultaneous positivity for internet addiction and elevated maladaptive cognitive emotion regulation.

#### 2.2.2 Predictor variables

We collected a set of independent characteristics encompassing sociodemographic factors and psychosocial resources as input features for the classification models.

We used a general demographic questionnaire to collect data on gender, age, educational attainment, marital status, and living with children.

We quantified subjective well-being using the Chinese version of the General Well-Being Scale (GWB), which was initially developed in the original study (26) and subsequently translated and adapted for Chinese populations (27). This 18-item inventory screens six dimensions of mental health: health concerns, energy, life satisfaction and interest, depressed or cheerful mood, emotional and behavioral control, and relaxation and tension. Scoring involves a mixture of 5-point and 6-point scales for items 1–14, and 0–10 scales for items 15–18. Notably, items 1, 3, 6, 7, 9, 11, 13, 15, and 16 require reverse scoring prior to summation. Total scores range from 14 to 120, with elevated numbers indicating superior well-being. The internal consistency (Cronbach’s alpha) of the GWB in this study was 0.829.

We explored family functioning dynamics via the Chinese version of the Family Adaptability and Cohesion Evaluation Scale (FACES), originally formulated in the initial study (28) and subsequently standardized for Chinese populations (29). The scale features 30 items evaluated on a 5-point frequency scale from 1 (never) to 5 (always), divided into two subscales: family cohesion (the emotional bonding among family members) and family adaptability (the system’s flexibility to modify structures in response to situational stress). The internal reliability coefficients (Cronbach’s alpha) were 0.927 for the total framework, 0.859 for family cohesion, and 0.866 for family adaptability.

We appraised the level of social assistance using the Social Support Rating Scale (SSRS) designed for use in China (30). This 10-item instrument yields an aggregate social support score derived from three foundational subscales: objective support (tangible, visible assistance received), subjective support (the psychological perception of being supported and cared for), and support utilization (the active behavior of seeking and utilizing available resources). Higher total scores reflect a more robust social support network for the older adult. In the current investigation, the Cronbach’s alpha coefficient for the total SSRS scale was 0.857.

### 2.3 Data preprocessing

**Figure 2:**
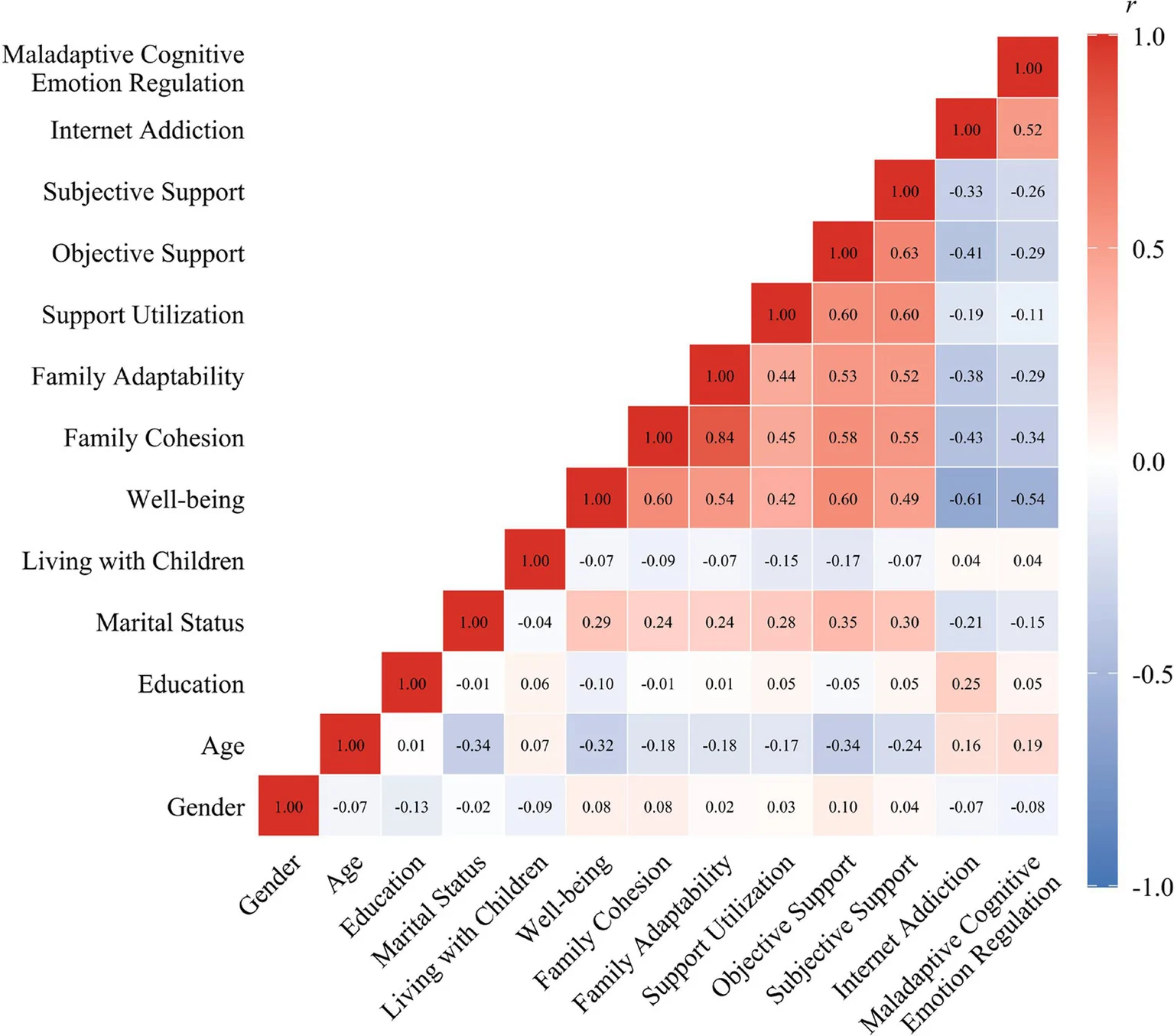
*Heatmap of feature correlations.* The heatmap shows pairwise Spearman correlation coefficients among the 13 included features, encompassing both the candidate predictors and the outcome variables (Internet Addiction and Maladaptive Cognitive Emotion Regulation). Color denotes correlation direction and magnitude (red: positive; blue: negative). Darker shades indicate stronger correlations, with the darkest red corresponding to *r* = 1.00 and the darkest blue corresponding to *r* = −1.00. The lower triangular portion displays the correlation coefficients using both numerical values and color gradients.

We initially included 11 candidate predictors from predefined individual-, family, and social-level variables. No variable exceeded the missingness threshold. First, we performed logical consistency checks to exclude implausible values. Second, within the training cohort, we identified statistical outliers using boxplots and the interquartile range (IQR). We handled outliers in the training cohort using winsorization to replace values beyond Q3 + 1.5 × IQR or below Q1–1.5 × IQR with the corresponding boundary values, as shown in Supplementary Figure S1 (Williamson et al., 1989). We then standardized continuous features using a *Z*-score transformation, where the scaling parameters (mean and standard deviation) were calculated strictly from the training cohort and subsequently applied unchanged to both cohorts, as shown in Supplementary Figure S2 (Andrade, 2021). To prevent data leakage, we determined the operational screening cutoff for elevated maladaptive cognitive emotion regulation in the training set by maximizing the Youden index via ROC analysis using internet addiction status as the reference outcome, yielding an optimal threshold of 37.50 (AUC = 0.822; Supplementary Figure S3). This data-driven threshold was treated as a cohort-specific operational screening cutoff rather than a clinical diagnostic criterion (31, 32). We used a two-step feature selection strategy to reduce feature redundancy. First, within the training cohort, we performed Spearman correlation analysis to identify highly correlated variables and excluded features with absolute correlation coefficients greater than 0.80. Second, within the training cohort, we implemented a 10-fold cross-validated LASSO regression incorporating inverse frequency weights to identify the most significant predictors for the model while mitigating class imbalance. We evaluated both *λ*.min and *λ*.1se. The *λ*.min criterion was selected for subsequent model training, retaining eight features, whereas *λ*.1se retained three features. Figure 2, Supplementary Figure S4, and Tables S1, S2 show detailed results of the feature selection procedure.

### 2.4 Machine learning construction and evaluation

In R, we developed and compared five machine learning models—LR, random forest (RF), SVM, GBM, and MLP—using the eight selected core features to identify the best-performing model. To achieve peak predictive performance and thoroughly mitigate class imbalance, algorithm-specific cost-sensitive learning frameworks were implemented during model training, utilizing an exhaustive grid search coupled with a five-fold cross-validation protocol to identify the optimal parameter spaces for all candidate estimators. We performed model fitting and hyperparameter tuning using cross-validation within the training set, while using the held-out test set for comparative internal evaluation of the five candidate models. We evaluated model performance across three dimensions: discrimination (measured by AUROC, accuracy, sensitivity, and specificity), calibration (quantified using Brier scores and calibration plots), and clinical utility (appraised via decision curve analysis [DCA]). We subsequently took the selected model forward for interpretability analysis.

### 2.5 Model interpretability analysis

To interpret the best-performing model, we used the SHAP framework (33). To improve the stability of Kernel SHAP estimation, we increased the number of sampling iterations to 100 and used a representative background dataset for resampling. The interpretability analysis included SHAP global importance bar plots, beeswarm plots, dependence plots, and force plots for representative cases with high and low model-predicted probabilities of current co-occurrence, thereby enabling both global and individual-level interpretation of model predictions (34).

### 2.6 Statistical analysis

We expressed categorical variables as counts and percentages. We described continuous data as mean ± SD or median (IQR), based on normality testing. We used chi-square and Wilcoxon rank-sum tests to compare categorical and continuous variables, respectively, and reported Cramer’s V and standardized mean differences (SMDs) as the corresponding effect-size measures. Results were regarded as statistically significant when the two-sided *p* value was below the 0.05 alpha level. We performed all analyses using R (v4.5.2). We generated statistical plots in R, prepared the workflow diagram in draw.io, and refined the final figure layout in Adobe Illustrator.

## 3 Results

### 3.1 Participant characteristics

After data cleaning, we included 1,471 participants in the final analysis. We divided the dataset into a training set of 1,030 participants (70.0%) and a test set of 441 participants (30.0%). Based on the predefined outcome criteria, 172 participants in the training set (16.70%) and 79 participants in the test set (17.91%) were classified into the co-occurrence group. In contrast, the remaining 858 (83.30%) and 362 (82.09%) participants were classified as controls, respectively. Tables 1, 2 present the baseline characteristics of participants in the training and test sets, stratified by co-occurrence status, incorporating Standardized Mean Differences (SMD) for continuous variables and Cramer’s V for categorical variables to report absolute effect sizes.

**Table 1:** Comparison of baseline characteristics of study participants in the training set.

Characteristics	Overall (*n* = 1,030)	Control (*n* = 858)	Co-occurrence Group (*n* = 172)	*p***-**value	Effect size
Well-being, M (P25, P75)	113.08 [99.91, 127.27]	117.65 [104.98, 130.31]	96.87 [91.80, 102.19]	<0.001	SMD: 1.1892
Family cohesion, M (P25, P75)	64.94 [57.93, 72.95]	66.94 [58.93, 75.95]	61.94 [56.93, 65.94]	<0.001	SMD: 0.5319
Family adaptability, M (P25, P75)	46.00 [40.00, 53.00]	48.00 [40.00, 55.00]	43.00 [39.00, 47.00]	<0.001	SMD: 0.3692
Objective support, M (P25, P75)	10.01 [8.02, 12.00]	10.01 [8.02, 12.00]	8.02 [6.03, 10.01]	<0.001	SMD: 0.7049
Subjective support, M (P25, P75)	11.94 [8.91, 13.95]	11.94 [9.92, 13.95]	10.93 [8.91, 11.94]	<0.001	SMD: 0.4127
Support utilization, M (P25, P75)	9.95 [6.99, 10.94]	9.95 [7.98, 10.94]	8.97 [6.99, 10.94]	0.006	SMD: 0.2325
Gender, *n* (%)				<0.001	Cramer’s V: 0.1090
1	468 (45.4%)	369 (43.0%)	99 (57.6%)		
2	562 (54.6%)	489 (57.0%)	73 (42.4%)		
Age, *n* (%)				<0.001	Cramer’s V: 0.1960
1	577 (56.0%)	518 (60.4%)	59 (34.3%)		
2	296 (28.7%)	223 (26.0%)	73 (42.4%)		
3	157 (15.2%)	117 (13.6%)	40 (23.3%)		
Education, *n* (%)				<0.001	Cramer’s V: 0.2161
1	391 (38.0%)	365 (42.5%)	26 (15.1%)		
2	305 (29.6%)	240 (28.0%)	65 (37.8%)		
3	211 (20.5%)	156 (18.2%)	55 (32.0%)		
4	123 (11.9%)	97 (11.3%)	26 (15.1%)		
Marital status, *n* (%)				<0.001	Cramer’s V: 0.1916
1	692 (67.2%)	611 (71.2%)	81 (47.1%)		
2	338 (32.8%)	247 (28.8%)	91 (52.9%)		
Living with children, *n* (%)				0.656	Cramer’s V: 0.0156
1	532 (51.7%)	440 (51.3%)	92 (53.5%)		
2	498 (48.3%)	418 (48.7%)	80 (46.5%)		

Continuous variables are presented as median (IQR) and compared groups using the Wilcoxon rank-sum test with Standardized Mean Differences (SMD) reported as effect sizes. We presented categorical variables as frequencies (percentages) and evaluated them using the Chi-square test with Cramer’s V reported as effect sizes.

**Table 2:** Comparison of baseline characteristics of study participants in the test set.

Characteristics	Overall (*n* = 441)	Control (*n* = 362)	Co-occurrence Group (*n* = 79)	*p-*value	Effect Size
Well-being, M (P25, P75)	111.80 [100.10, 128.37]	120.57 [105.95, 131.29]	95.23 [91.82, 99.13]	<0.001	SMD: 1.4274
Family cohesion, M (P25, P75)	66.13 [58.13, 74.13]	68.13 [59.13, 76.13]	61.13 [57.13, 65.13]	<0.001	SMD: 0.6137
Family adaptability, M (P25, P75)	47.00 [40.00, 53.00]	48.00 [41.00, 54.00]	42.00 [38.00, 47.00]	<0.001	SMD: 0.4703
Objective support, M (P25, P75)	9.98 [7.95, 12.00]	9.98 [7.95, 12.00]	7.95 [5.93, 9.98]	<0.001	SMD: 0.7271
Subjective support, M (P25, P75)	12.15 [9.20, 14.12]	12.15 [10.18, 14.12]	11.16 [8.21, 13.13]	<0.001	SMD: 0.4033
Support utilization, M (P25, P75)	9.08 [7.02, 11.15]	9.08 [8.05, 11.15]	9.08 [7.02, 11.15]	0.051	SMD: 0.2499
Gender, *n* (%)				0.150	Cramer’s V: 0.0744
1	205 (46.5%)	162 (44.8%)	43 (54.4%)		
2	236 (53.5%)	200 (55.2%)	36 (45.6%)		
Age, *n* (%)				<0.001	Cramer’s V: 0.2310
1	253 (57.4%)	227 (62.7%)	26 (32.9%)		
2	135 (30.6%)	97 (26.8%)	38 (48.1%)		
3	53 (12.0%)	38 (10.5%)	15 (19.0%)		
Education, *n* (%)				<0.001	Cramer’s V: 0.2042
1	183 (41.5%)	167 (46.1%)	16 (20.3%)		
2	123 (27.9%)	95 (26.2%)	28 (35.4%)		
3	79 (17.9%)	58 (16.0%)	21 (26.6%)		
4	56 (12.7%)	42 (11.6%)	14 (17.7%)		
Marital Status, *n* (%)				<0.001	Cramer’s V: 0.1901
1	306 (69.4%)	266 (73.5%)	40 (50.6%)		
2	135 (30.6%)	96 (26.5%)	39 (49.4%)		
Living with children, *n* (%)				0.475	Cramer’s V: 0.0399
1	238 (54.0%)	192 (53.0%)	46 (58.2%)		
2	203 (46.0%)	170 (47.0%)	33 (41.8%)		

Continuous variables are presented as median (IQR) and compared groups using the Wilcoxon rank-sum test with Standardized Mean Differences (SMD) reported as effect sizes. We presented categorical variables as frequencies (percentages) and evaluated them using the Chi-square test with Cramer’s V reported as effect sizes.

### 3.2 Feature selection and retention

To identify features relevant to classifying current co-occurrence status and optimize model performance, this study employed a two-step feature selection strategy. First, Spearman correlation analysis and collinearity assessment of the 11 initial features revealed high collinearity between family cohesion and family adaptability (*r* = 0.84), as shown in Figure 2. Based on clinical relevance and theoretical considerations, we excluded family adaptability and retained family cohesion for subsequent analysis. Notably, the correlation between general well-being and maladaptive cognitive emotion regulation was only moderate (*r* = −0.54), demonstrating a clear absence of statistical redundancy or information overlap between the dominant predictor and the outcome. After this step, the remaining 10 features met the multicollinearity criteria (variance inflation factor [VIF] < 5).

**Figure 3:**
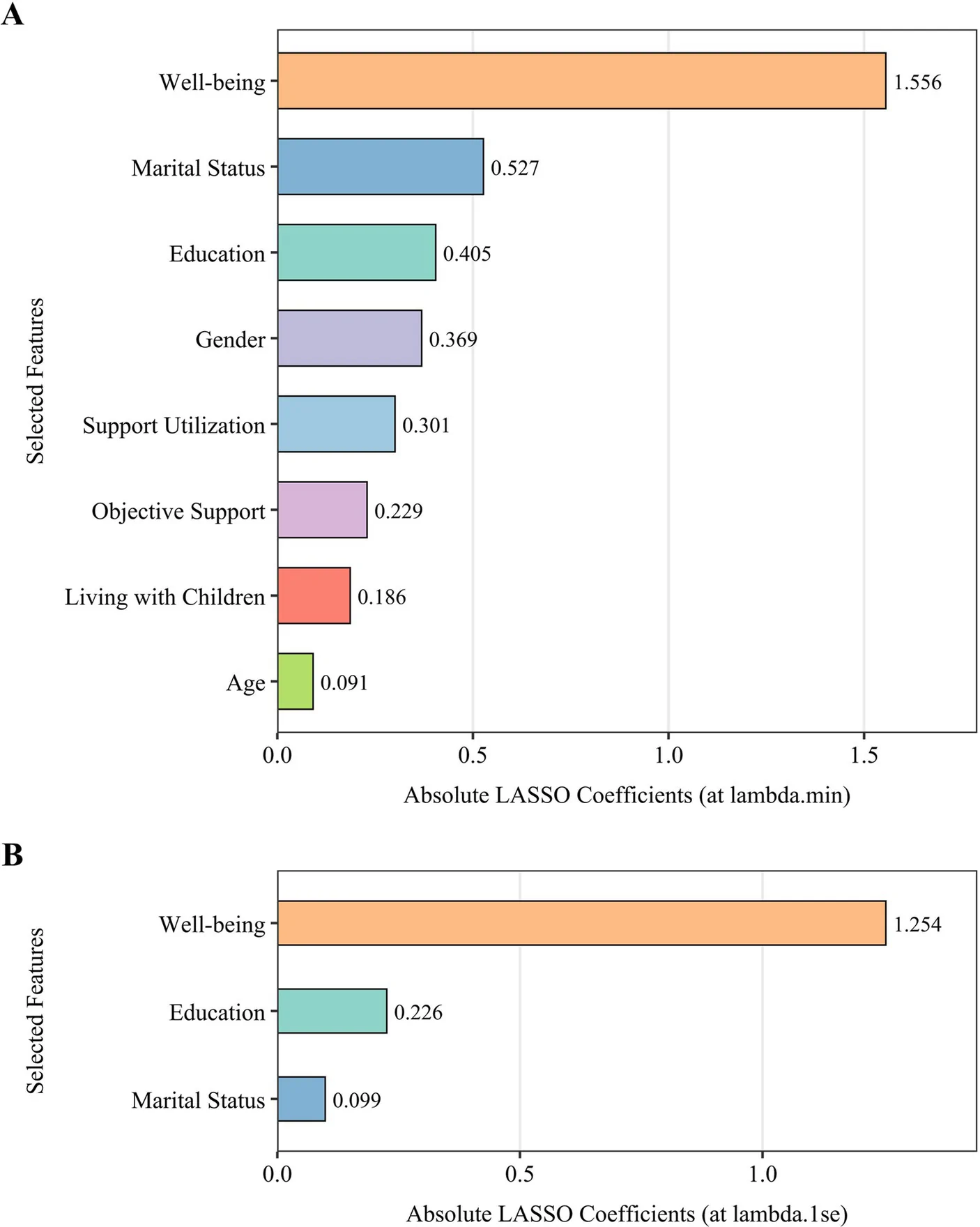
Absolute LASSO coefficients of the retained features at *λ*.min and *λ*.1se. The bar plots illustrate the absolute values of the LASSO coefficients for features selected under different regularization thresholds. **(A)** Features retained under the *λ*.min criterion, yielding eight selected predictors. **(B)** Features retained under the *λ*.1se criterion, demonstrating enhanced model parsimony with three predictors. Different colors represent different features. Higher absolute values reflect a greater relative weight of the corresponding feature in the regularization model.

Second, we applied LASSO regression for dimensionality reduction and feature selection. We entered the 10 retained features into the model, and the penalty parameter *λ* enabled automatic variable selection and regularization. We used ten-fold cross-validation to determine the optimal regularization parameter. We evaluated the regularization path where *λ*.min = 0.00687 and *λ*.1se = 0.04419. To ensure a robust predictor set, the *λ*.min criterion was selected, retaining eight core features. Figures 3A,B present the absolute LASSO coefficients for the eight retained features at *λ*.min and the three retained features at *λ*.1se, providing an intuitive summary of their relative contributions to feature selection. As shown in Supplementary Figure S4A, the regularization path indicated that, as *λ* increased, coefficients for features with lower contributions were progressively shrunk toward zero, thereby improving model sparsity. Supplementary Figure S4B shows the cross-validation results, in which the mean deviance reached its minimum at *λ*.min = 0.00687. *λ*.1se was 0.04419 and remained within one standard error of the minimum. Ultimately, we retained eight core predictors for subsequent model training: general well-being, support utilization, age, gender, educational attainment, marital status, objective support, and living with children, as listed in Supplementary Table S1.

### 3.3 Multi-model establishment and validation

**Figure 4:**
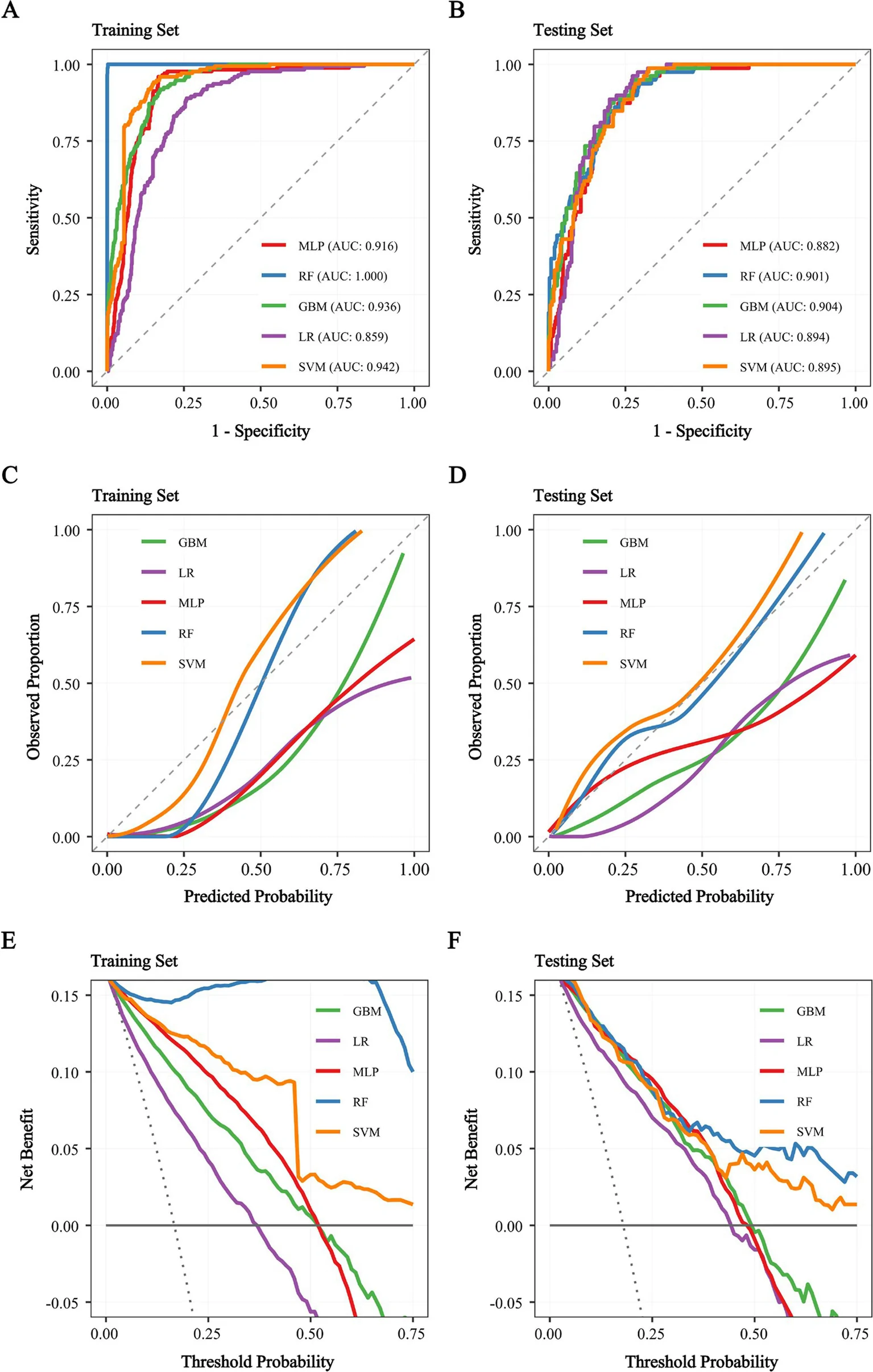
Performance evaluation curves for different machine learning models. Different colored lines represent different machine learning algorithms. Panels **(A,C,E)** display the metrics calculated within the training cohort, while panels **(B,D,F)** show the corresponding evaluation within the held-out test cohort. The receiver operating characteristic (ROC) curves evaluate the discriminative performance of the candidate algorithms, with the Area Under the Curve (AUC) values listed in the legends quantifying global classification accuracy. **(A)** ROC curves in the training cohort. **(B)** ROC curves in the held-out test cohort, where the gradient boosting machine (GBM) achieved the optimal testing AUROC of 0.904. **(C)** Calibration curves in the training cohort, illustrating the agreement between predicted and observed probabilities. **(D)** Calibration curves in the held-out test cohort. **(E)** Decision Curve Analysis (DCA) in the training cohort, showing the clinical net benefit across different threshold probabilities. **(F)** DCA curves in the held-out test cohort.

**Figure 5:**
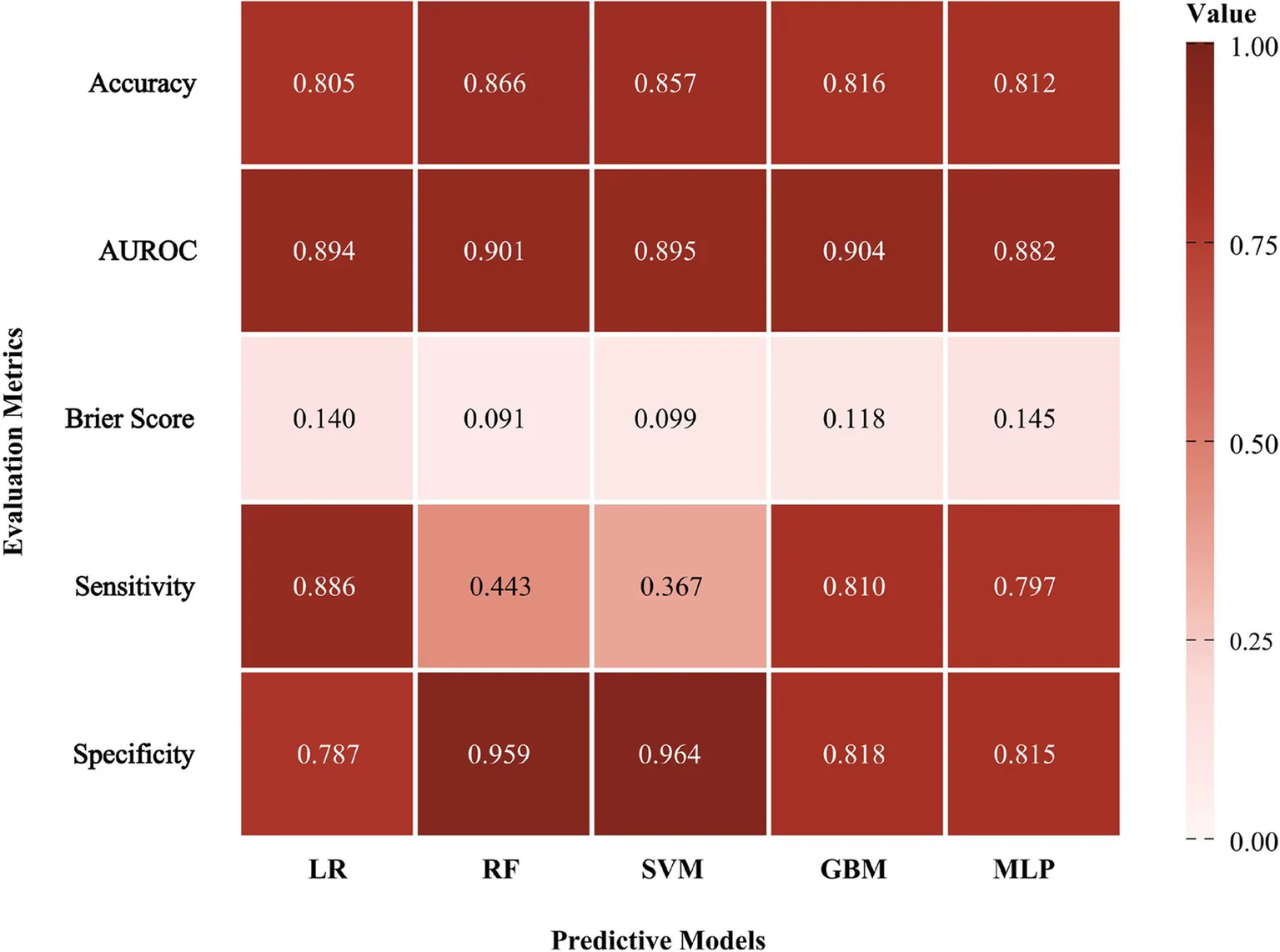
Heatmap comparing the performance of different machine learning models on the test set. The heatmap presents multiple evaluation metrics across the candidate models. The horizontal axis represents the predictive models, and the vertical axis displays the evaluation metrics (Accuracy, AUROC, Brier Score, Sensitivity, and Specificity). Darker red shades indicate values closer to 1.00, and numerical labels indicate the specific performance values for each model.

Using the eight LASSO-selected features (general well-being, support utilization, age, gender, educational attainment, marital status, objective support, and living with children), we developed five parallel machine-learning models in the training set and evaluated them in the held-out test set. Although RF and SVM achieved high accuracies, their sensitivities were comparatively low (0.443 and 0.367, respectively). In contrast, the GBM model demonstrated the most balanced overall performance (AUROC = 0.904, accuracy = 0.816, Brier score = 0.118, sensitivity = 0.810, specificity = 0.818). Consequently, GBM was selected as the optimal classifier for identifying co-occurrence status among older adults, with discrimination, calibration, and decision curves presented in Figures 4A–F and comparative test-set performance metrics summarized in Figure 5.

### 3.4 Model interpretation and deployment

**Figure 6:**
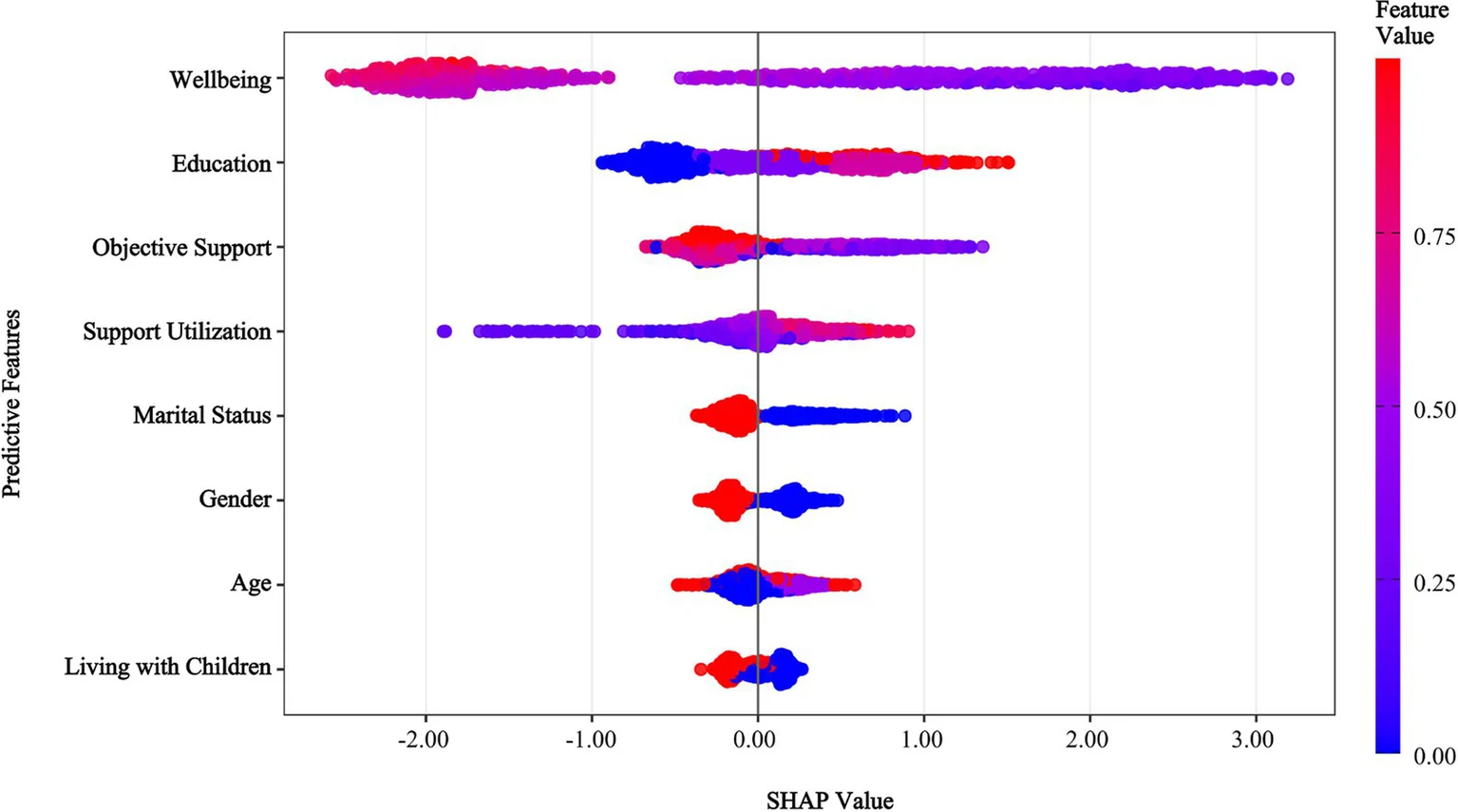
SHAP-based interpretation of feature effects in the GBM model. The beeswarm plot illustrates the distribution and directional impact of SHAP values for each feature across all samples, with predictors ranked from top to bottom by their average absolute SHAP magnitude to reflect global importance. Point colors indicate feature values from low (blue) to high (red). Positions to the right of the vertical zero reference line signify a positive impact on the outcome, whereas points to the left indicate a negative contribution.

Based on the optimal GBM model, we performed a multi-level interpretability analysis using SHAP. Figure 6 shows the model’s global interpretation results. The SHAP summary plot indicated that general well-being, educational attainment, objective support, support utilization, marital status, gender, age, and living with children were the eight predictors, ranked by the magnitude of their cumulative contributions. General well-being showed substantially larger aggregated SHAP values than the other features, indicating that it was the most influential feature in the model. The SHAP beeswarm plot further illustrated the directional effects of these features on model predictions. General well-being and being married or partnered were associated with a lower model-predicted probability of current co-occurrence. Conversely, higher educational attainment, older age, and male gender were associated with a higher model-predicted probability of current co-occurrence, whereas support utilization showed a nonlinear and context-dependent contribution to model predictions. Together, these findings suggest potentially complex joint patterns among psychosocial and demographic features in the model.

**Figure 7:**
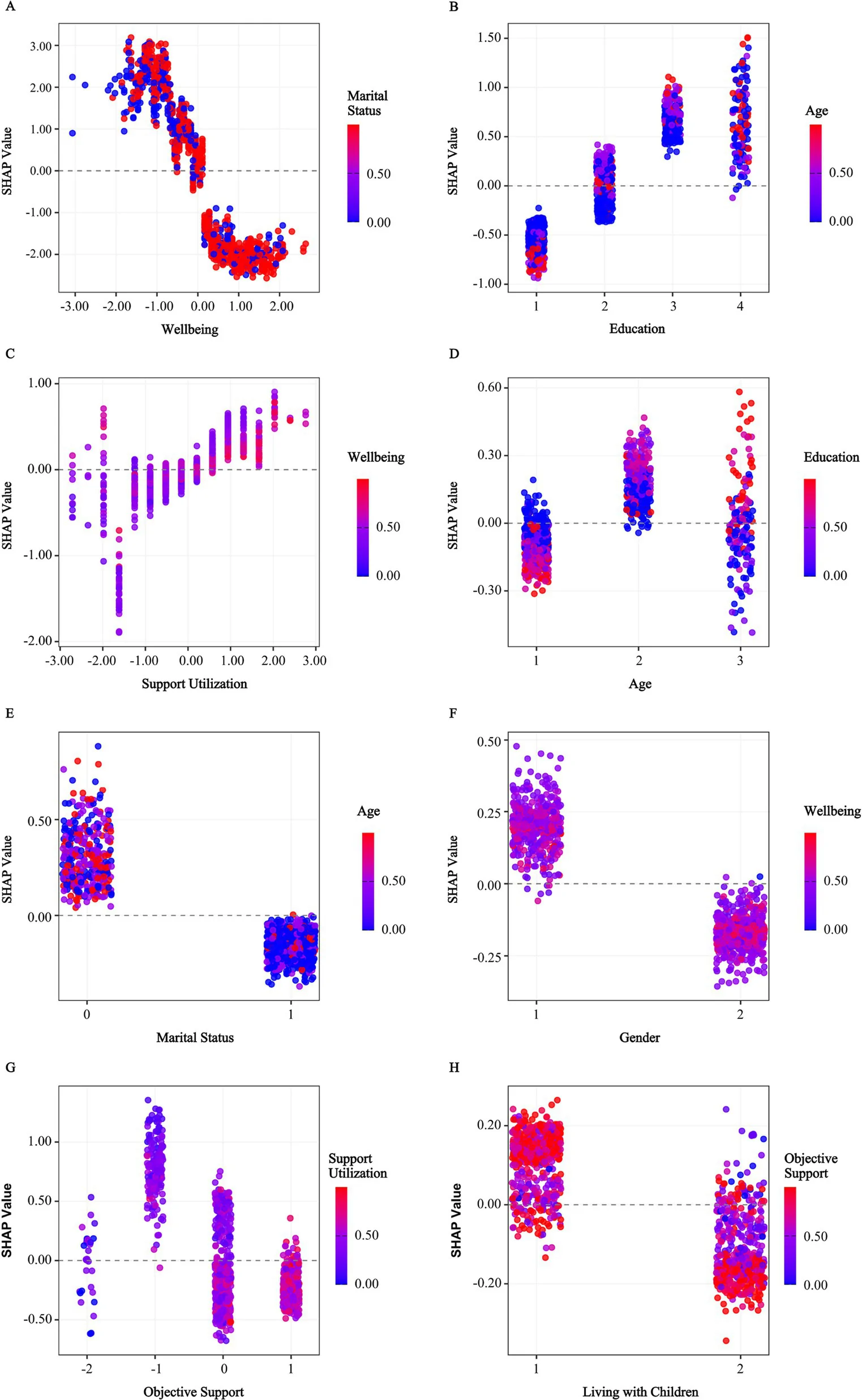
Analysis of pairwise feature patterns based on SHAP dependence plots. SHAP dependence plots illustrate the joint patterns of two features in relation to the model-predicted probability of current co-occurrence. The panels show interactions between the following feature pairs: **(A)** Well-being and marital status; **(B)** Education and age; **(C)** Support utilization and well-being; **(D)** Age and education; **(E)** Marital status and age; **(F)** Gender and well-being; **(G)** Objective support and support utilization; **(H)** Living with children and objective support.

Figure 7 shows the pairwise interaction patterns identified by the SHAP dependence plots. General well-being was negatively associated with the model-predicted probability of current co-occurrence, with a change in the SHAP pattern around 105 points. Scores above this value were generally associated with a lower predicted probability, whereas lower scores were associated with a higher predicted probability. This association appeared to be more pronounced among participants who were married or partnered. Support utilization showed a nonlinear SHAP pattern, with a change in model output around 10 points. Notably, the model-predicted probability of current co-occurrence was highest when high support utilization co-occurred with low general well-being.

Age and educational attainment also showed nonlinear joint patterns in model predictions, with the contribution of each feature varying across levels of the other. The interaction between gender and general well-being revealed a higher predicted probability among men with low well-being. When examined independently, educational attainment showed a generally increasing but nonlinear pattern across categories. Joint analysis of marital status and age further indicated that younger partnered participants had the lowest predicted probability, suggesting a combined association of social connection and life stage with lower predicted probability. Objective support showed a nonlinear SHAP pattern, with the strongest positive contributions observed around a standardized value of −1, whereas higher standardized values were generally associated with negative SHAP contributions; this pattern varied with support utilization. Participants living with children generally showed positive SHAP values, whereas those not living with children generally showed negative SHAP values, with the magnitude varying across levels of objective support.

**Figure 8:**
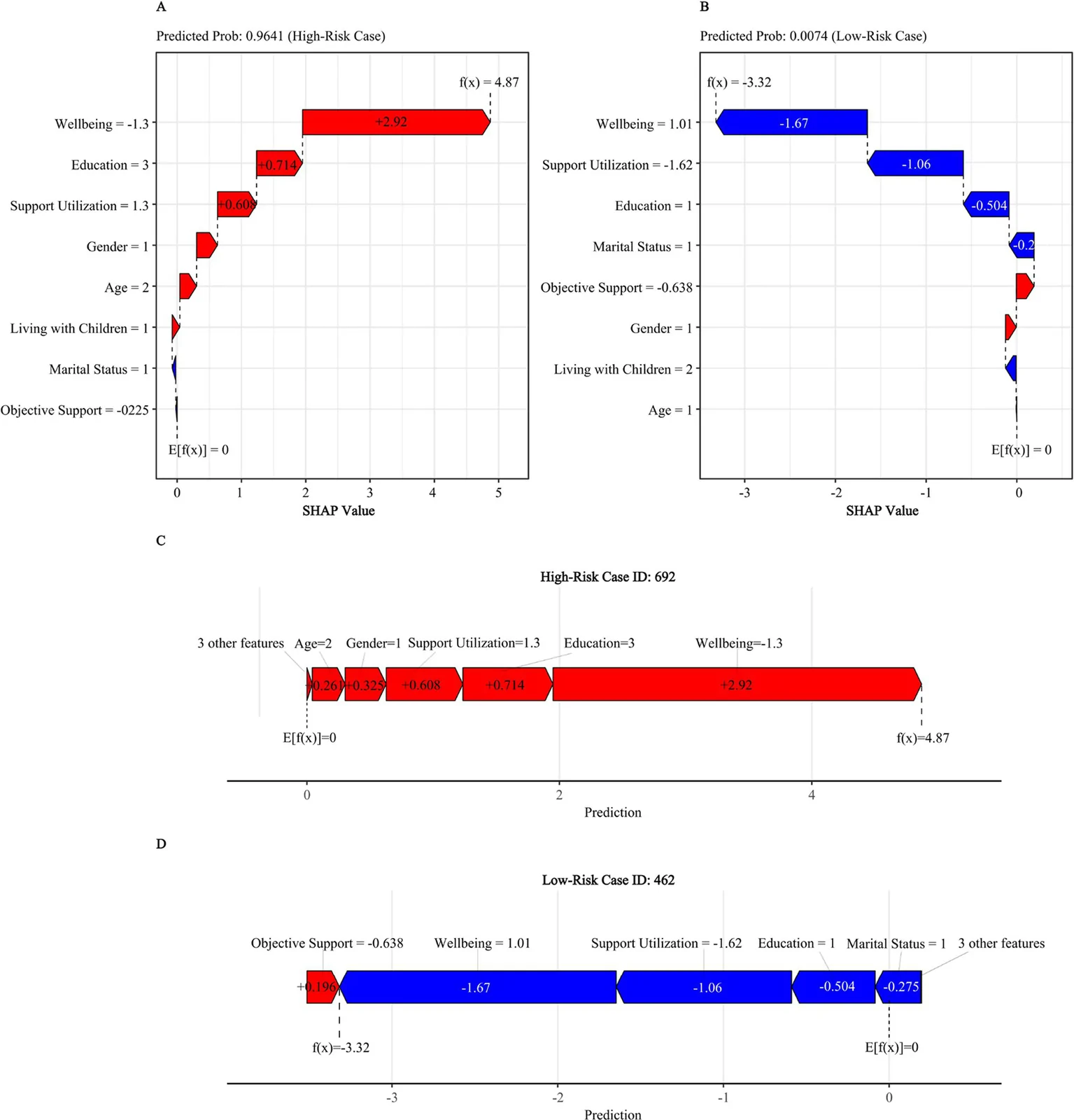
SHAP force and waterfall plot analysis of representative individual cases. Force plots and waterfall plots illustrate the sign and intensity of feature contributions on the final model output for individual samples. Features that push the model output to the right (in force plots) or in a positive direction (in waterfall plots) increase the model-predicted probability of current co-occurrence, whereas features that push it in the opposite direction decrease this probability. The length of each segment reflects the magnitude of its contribution. The base value, *E*[*f*(*x*)], represents the baseline model output, and *f*(*x*) denotes the final log-odds prediction for the individual sample. **(A)** Waterfall plot for a high-risk case (Case ID: 692). **(B)** Waterfall plot for a low-risk case (Case ID: 462). **(C)** Force plot for the high-risk case (Case ID: 692). **(D)** Force plot for the low-risk case (Case ID: 462).

Figure 8 shows local SHAP explanations for representative cases with low and high model-predicted probabilities of current co-occurrence. Figures 8A and 8B present waterfall plots for cases with high and low model-predicted probabilities, respectively. In the case with a low predicted probability, being unpartnered and male contributed to a higher predicted probability, whereas general well-being, educational attainment, age, and support utilization contributed to a lower predicted probability of current co-occurrence. In the case with a high predicted probability, general well-being, support utilization, educational attainment, age, and marital status contributed to a higher predicted probability, whereas gender contributed to a lower predicted probability. Figures 8C and 8D present force plots for the same representative cases, providing a complementary visualization of the direction and magnitude of individual feature contributions to the final prediction.

To provide an accessible demonstration of the classification model, this study developed an interactive Shiny web application. Using the shiny package in R, we implemented the selected GBM model to provide real-time classification results and visual explanations. The online application is available at https://guijingrui.shinyapps.io/elderly-ia-emotion-risk/, and the source code has been deposited on GitHub at https://github.com/Mortyandy/elderly-ia-emotion-risk.git to facilitate secondary development and localized adaptation. This application provides an exploratory interface for identifying current co-occurrence and visualizing individual-level feature contributions.

## 4 Discussion

Increasing internet use among older adults has accompanied the widespread adoption of digital technology. However, older adults facing cognitive decline and social isolation may struggle to regulate emotions effectively during extended internet use. Maladaptive cognitive emotion regulation strategies, such as rumination and catastrophizing, may not only prolong psychological distress but also increase vulnerability to adverse mental health outcomes, including depression, anxiety, and suicidality. Therefore, developing an interpretable classification model may help identify older adults with current co-occurring internet addiction and elevated maladaptive cognitive emotion regulation and characterize the cross-sectional features associated with this co-occurrence.

### 4.1 Core findings and analysis of counterintuitive model patterns

This study characterized the cross-sectional co-occurrence pattern of internet addiction and elevated maladaptive cognitive emotion regulation among older adults, identified influential model features including general well-being and support utilization, and developed a machine-learning classifier that balanced classification performance and interpretability. The co-occurrence and control groups differed significantly in gender, age, educational attainment, marital status, and multiple psychosocial indicators, which is broadly consistent with previous studies (35, 36). General well-being emerged as the most influential feature in the classifier, with higher scores generally associated with a lower model-predicted probability of current co-occurrence. The dependence plot suggested a change in the model pattern around 105 points. In addition, the association between higher well-being and lower predicted probability appeared more pronounced among partnered participants, whereas unpartnered participants showed a steeper increase in predicted probability at lower well-being levels. This pattern indicates that the association between general well-being and the model-predicted probability of current co-occurrence differed by marital status.

In this study, we can interpret the higher proportion of unpartnered older adults in the co-occurrence group through multiple theoretical lenses. According to the Uses and Gratifications Theory, when emotional needs remain unmet, particularly among widowed or solitary older adults, the internet may serve as an actively sought compensatory outlet (37). Prolonged compensatory internet use may be associated with more persistent maladaptive cognitive-emotional regulation patterns, a possibility that warrants examination in future research (38). Cognitive Emotion Regulation Strategies Theory further suggests that insufficient external support heightens vulnerability to rigid, maladaptive coping strategies when managing negative emotions (39). Self-Determination Theory also offers a relevant explanation, as unmet needs for relatedness, stemming from insufficient real-world support, may foster compensatory internet engagement (40). As offline support diminishes, the co-occurrence of internet dependence and maladaptive regulation may become more pronounced, raising the hypothesis of a reciprocal association that should be examined in future research (41).

A particularly noteworthy finding was the counterintuitive association between support utilization and the model-predicted probability of current co-occurrence. Whereas traditional social support theory generally emphasizes the beneficial role of support resources, the dependence plot suggested a nonlinear pattern around 10 points, above which the predicted probability tended to increase. This pattern differs from the conventional expectation that greater social support is generally associated with more favorable outcomes. The unadjusted group comparisons and the SHAP results address different questions. The former summarize the overall distribution of support utilization between groups, whereas the latter describe its conditional contribution within the multivariable model. Therefore, the apparent discrepancy may reflect a nonlinear and context-dependent relationship, particularly at lower levels of general well-being. One possible interpretation is that this pattern may be related to an imbalance between support demand and available resources. Accumulated stress in later life may heighten emotion regulation demands; when adaptive resources are insufficient, maladaptive strategies may be adopted as compensatory substitutes (35). Accordingly, higher support utilization should not be interpreted as a uniformly adverse factor. One possible explanation is that, among participants with lower well-being, greater support utilization may reflect elevated support needs rather than effective support receipt; however, this interpretation remains speculative (38, 40). This finding carries important implications for community intervention, suggesting that merely increasing support quantity may prove insufficient; improving the quality and appropriateness of support may be more critical.

From the perspective of Self-Determination Theory, frustration of autonomy, competence, and relatedness needs in later life may render the internet a low-cost compensatory channel for unmet psychological needs. Following reduced social role engagement, older men are particularly inclined to seek autonomy and competence fulfillment through internet-mediated activities (42). The internet’s low accessibility threshold and high interactivity may provide rapid positive reinforcement, potentially accelerating the transition from casual use to compulsive engagement (43, 44). When coupled with maladaptive cognitive emotion regulation, it may further increase the predicted risk of co-occurrence (45, 46). Regarding age differences, young-old adults (60–70 years) predominated in the co-occurrence group. One possible interpretation is that this group may possess relatively greater cognitive and learning capacities and internet access, while also experiencing major role transitions, such as retirement, empty-nest experiences, and health-related anxiety. In the absence of adaptive emotion regulation strategies, they may therefore be particularly susceptible to internet-based emotional escape, consistent with the Stress-Coping Theory formulations linking passive coping to addictive behaviors. Notably, higher educational attainment, which typically confers a technological advantage, may paradoxically increase vulnerability when experiencing role deprivation and a loss of purpose. In the absence of effective real-world channels for emotional adjustment, higher digital literacy may facilitate compensatory online engagement (1, 46, 47). This interpretation raises the hypothesis that digital capacity, perceived deprivation, compensatory internet use, and dependence may be linked, which should be examined in future research.

### 4.2 SHAP dependence patterns and potential interpretations

By incorporating SHAP, this study sought to address the “black box” nature of machine learning models. SHAP dependence analysis further showed nonlinear changes in model output for several features. First, the associations of external resource-related features with model output appeared to vary across levels of general well-being. The associations of partner relationships and social support with a lower model-predicted probability became more evident at higher levels of general well-being. In contrast, elevated support utilization was associated with a higher model-predicted probability under conditions of low well-being. This pattern may be consistent with an imbalance between internal and external resources (48). One possible explanation is that low general well-being may reflect limited internal psychological resources, although this mechanism was not directly examined. SHAP analysis showed that higher educational attainment and elevated support utilization ranked among the most influential model features. The prominence of these features may be consistent with the potential roles of digital capacity use and internal–external resource imbalance in current co-occurrence patterns, although these mechanisms were not directly examined (1, 35). Although age, gender, and marital status contributed less strongly at the global level, they showed joint patterns with psychological variables, suggesting that demographic context may influence the model-predicted probability of current co-occurrence.

Secondly, age and educational attainment showed a nonlinear joint pattern in model predictions. Higher educational attainment and older age were jointly associated with a higher model-predicted probability; however, an inflection point at the university level or above coincided with a slight decline in the model-predicted probability (49). The data further showed that, within the highest educational category, the model-predicted probability declined modestly. This finding suggests that a high level of education may confer greater cognitive reserve and executive control, thereby partially buffering addiction-related risks associated with excessive internet exposure (47, 50). In addition, the interaction analysis suggested that positive psychological capital was associated with lower model-predicted probabilities across demographic groups. Although the preceding findings indicated higher baseline risk among men and young-old adults aged 60–70 years, high general well-being appeared to weaken the maladaptive coping disadvantage associated with these groups (51–53). Similarly, stable partner relationships may function as an important emotional anchor, partly offsetting the elevated transitional risks associated with the retirement period among young-old adults (35, 54).

Thirdly, the findings further highlight the role of psychological capital in model patterns associated with external resources. The dependence plots further suggested that the model contributions of objective support and living with children varied according to support utilization and objective support, respectively. Building on the preceding discussion of partner relationships and social support, the results suggest that the associations of external network resources with lower model-predicted probabilities may vary according to psychological context and appear more clearly when higher intrinsic well-being accompanies partner relationships (55, 56). By contrast, when general well-being was extremely low, greater support utilization was associated with a higher model-predicted probability. This counterintuitive pattern may reflect the internal–external resource imbalance discussed above, although the underlying mechanism was not directly examined in the present study (57, 58). Taken together, these findings indicate a nonlinear joint pattern involving educational attainment, age, psychological capital, and external support resources, and further show that the combination of higher educational attainment and older age was associated with a higher model-predicted probability of current co-occurrence under certain conditions (40, 50).

### 4.3 Methodological innovations and limitations

Furthermore, the cohort-specific Youden index threshold was used as an operational screening cutoff rather than a clinical diagnostic criterion. Its generalizability requires further evaluation and recalibration in independent populations (31). Unlike previous studies that have relied primarily on univariate analyses to screen risk factors, this study used Spearman correlation analysis to assess multicollinearity, followed by LASSO regression for feature selection. This approach helped reduce overfitting and identify the core features most closely associated with co-occurrence, thereby improving model parsimony and stability within the current sample. By comparing five machine-learning algorithms in parallel, this study evaluated both linear and nonlinear classification approaches. The GBM model, with its capacity to capture complex nonlinear interactions and multi-way decision boundaries, achieved the best overall performance in the held-out test set. Notably, the inclusion of eight core predictors after feature selection, combined with the overall sample size of 1,471, provided an optimized sample-to-feature ratio to support robust algorithmic training. These findings suggest that, under conditions of moderate sample size and optimized feature selection (60), the developed GBM model (AUROC = 0.904) achieved high discriminatory capacity relative to classification models in geriatric mental health research (15, 36), suggesting that moderate sample sizes with optimized feature selection can support robust machine learning applications in aging populations.

The performance observed in this study may also reflect the particular challenges of prediction in complex co-occurrence contexts. One possible explanation for the observed performance differences lies in the intrinsic distinctions between outcome definitions. Compared with smartphone dependence, which is characterized primarily by a single behavioral dimension, the co-occurrence of internet addiction and elevated maladaptive cognitive emotion regulation examined in this study involves a more complex combination of psychological and behavioral characteristics. Additionally, differences in feature composition may also constrain the upper limit of model performance. Although the GBM demonstrated optimal predictive performance in this study, tree-based ensemble models remain more prediction-oriented than explanation-oriented. Therefore, further efforts are still needed to improve their clinical translatability and practical applicability.

### 4.4 Exploratory application and potential use scenarios

Building on the individual-level model explanations, this study developed and deployed an interactive web-based classification interface (a Shiny application) using the optimal GBM model. We developed this platform as an exploratory demonstration of how the classification model and its individual-level explanations could be presented in an accessible format. The interface provides a visual presentation of the model-predicted probability of current co-occurrence and the corresponding individual-level feature contributions. At this stage, the application is intended primarily for research demonstration and hypothesis generation rather than clinical screening or decision-making. Its practical utility requires further prospective evaluation and external validation in independent populations (33, 59).

### 4.5 Limitations

Despite providing meaningful insights into the classification and interpretation of the co-occurrence of internet addiction and elevated maladaptive cognitive emotion regulation in older adults, this study has several limitations. Firstly, the sample was recruited primarily from Anhui Province, China, which may have introduced geographic bias and may limit the generalizability of the findings to populations with different socioeconomic backgrounds or cultural contexts. Given the heterogeneity in digital infrastructure and the allocation of care resources for older adults across Chinese provinces, future research should conduct large-scale, multi-regional, and multicenter collaborative surveys to improve the external validity and generalizability of classification models. Although a held-out test set was used for internal validation, the model has not yet been externally validated in an independent geographic or temporal cohort. Secondly, this study used a cross-sectional design, with all model features and the co-occurrence outcome assessed within the same survey wave. Consequently, the reported model performance reflects the classification of current co-occurrence status rather than the prospective prediction of future onset. The temporal ordering and directionality of the observed associations could not be determined, and reverse causation cannot be excluded. Therefore, the identified features should be interpreted as contemporaneous correlates and contributors to model classification rather than causal or prospective risk factors. Future longitudinal studies with repeated assessments are required to evaluate whether these features predict incident co-occurrence over time. Finally, model inputs relied primarily on self-report questionnaires, which may be subject to measurement error related to subjective cognitive bias or social desirability. Future studies could integrate multimodal data, such as physiological signals and objective traces of internet use behavior, to construct more objective and higher-fidelity input features.

## 5 Conclusion

This study developed an interpretable machine-learning classifier for the co-occurrence of internet addiction and elevated maladaptive cognitive emotion regulation in older adults, using eight retained features: general well-being, support utilization, age, gender, educational attainment, marital status, objective support, and living with children. Among the five machine-learning models, the GBM showed the best overall classification performance. SHAP analysis provides a framework for describing feature contributions to the classification results. General well-being emerged as the most influential feature in the classifier, whereas older age, male gender, and higher educational attainment, as well as greater support utilization under conditions of lower general well-being were associated with a higher model-predicted probability of current co-occurrence, suggesting potentially counterintuitive associations between resource-related features and model output.

From a methodological perspective, this study enhances the interpretability of classification models by employing SHAP values to characterize individual-level feature contributions and joint patterns across features, thereby making model outputs more transparent and easier to interpret. From an exploratory translational perspective, the Shiny application presents model classification results at the individual level. Clarifying the major features contributing to the model-predicted probability of current co-occurrence may support the further evaluation of potential practical applications. From a public health perspective, this approach provides an exploratory framework for presenting current co-occurrence classifications, although prospective evaluation and external validation are required before clinical use, future-risk assessment, or preventive decision-making.

## Data Availability

The raw data supporting the conclusions of this article will be made available by the authors, without undue reservation.
