# Supplementary material for: Interpretable machine learning for identifying co-occurring internet addiction and maladaptive cognitive emotion regulation in older adults: a cross-sectional study
Source: Front Public Health. 2026 Aug 13;14:1901765. doi: 10.3389/fpubh.2026.1901765 (PMC13518514; doi:10.3389/fpubh.2026.1901765)
Supplement: Supplementary file 1 [file Supplementary_file_1.docx]

**Supplementary Material**

**Interpretable Machine Learning for Identifying Co-occurring Internet Addiction and Maladaptive Cognitive Emotion Regulation in Older Adults: A Cross-Sectional Study**

Wenjuan Wang, Ph.D

Bengbu Medical University, Bengbu, Anhui, 233030, China

Email: [wwj.wen@163.com](mailto:wwj.wen@163.com)

**This file includes:**

- **Table S1:** **Predictors retained under the λ.min criterion.**

**- Table S2: Predictors retained under the λ.1se criterion**

- **Figure S1:** Boxplots of continuous features before and after outlier processing

- **Figure S2:** Boxplots of continuous features before and after standardization

- **Figure S3:** ROC curve for determining the optimal cutoff for maladaptive cognitive emotion regulation based on the training set.

- **Figure S4:** LASSO coefficient path and cross-validation results.

**Table S1. Predictors retained under the λ.min criterion.**

| **Predictors** | **OR** | **95% CI** | **P Value** |
| --- | --- | --- | --- |
| Well-being | 0.1934 | 0.15-0.2495 | <0.001 |
| Marital Status | 0.5209 | 0.3658-0.7417 | <0.001 |
| Education | 1.5656 | 1.3278-1.8461 | <0.001 |
| Gender | 0.6312 | 0.4581-0.8697 | 0.005 |
| Support Utilization | 1.5199 | 1.2368-1.8677 | <0.001 |
| Objective Support | 0.7209 | 0.5784-0.8986 | 0.004 |
| Living with Children | 0.7457 | 0.5312-1.0468 | 0.090 |
| Age | 1.1076 | 0.8898-1.3787 | 0.360 |

Note. The table shows the predictors with non-zero coefficients selected by LASSO regression via 10-fold cross-validation (λ.min = 0.00687). These retained features were subsequently entered into a multivariable logistic regression model to obtain unpenalized statistical inferences and facilitate clinical interpretation. Data are presented as odds ratios (ORs), 95% confidence intervals (CIs), and p-values.

**Table S2. Predictors retained under the λ.1se criterion.**

| **Predictors** | **OR** | **95% CI** | **P Value** |
| --- | --- | --- | --- |
| Well-being | 0.1836 | 0.1453-0.2321 | <0.001 |
| Marital status | 0.5944 | 0.4323-0.8173 | 0.001 |
| Education | 1.6622 | 1.4144-1.9535 | <0.001 |

Note. The table shows the predictors with non-zero coefficients selected by LASSO regression via 10-fold cross-validation (λ.1se = 0.04419). This 1-standard-error criterion yields the most parsimonious model. These retained features were subsequently entered into a multivariable logistic regression model to obtain unpenalized statistical inferences and facilitate clinical interpretation. Data are presented as odds ratios (ORs), 95% confidence intervals (CIs), and p-values.

**Figure S1. Boxplots of continuous features before and after outlier processing.**

Note. The boxplots show the distributions of continuous features before and after outlier processing. The upper and lower boundaries of each box represent the third quartile (Q3) and first quartile (Q1), respectively. Red dots beyond the whiskers indicate outliers, defined as values greater than Q3 + 1.5 × IQR or less than Q1 − 1.5 × IQR. For the objective support feature, no upper whisker was observed before outlier processing, indicating that the maximum value was equal to Q3. (A) Before outlier processing. (B) After outlier processing.

**Figure S2. Boxplots of continuous features before and after standardization.**

Note. The boxplots show the distributions of continuous features before and after standardization. After standardization, all variables have a mean of 0 and a standard deviation of 1, which improves comparability across features while preserving their overall distributional patterns. (A) Before standardization. (B) After standardization.

**Figure S3. ROC curve for determining the optimal cutoff for maladaptive cognitive emotion regulation strategies based on the training set.**

Note. The receiver operating characteristic (ROC) curve illustrates the determination of the optimal operational cutoff for maladaptive cognitive emotion regulation. The red dot on the curve indicates the maximum Youden index (calculated as sensitivity + specificity - 1), identifying the optimal cutoff of 37.5 to balance sensitivity and specificity. At this threshold, the model achieved an Area Under the Curve (AUC) of 0.822, with a sensitivity of 1.000 and a specificity of 0.561.

**Figure S4. LASSO coefficient path and cross-validation results.**

Note. (A) Coefficient path plot. Color-coded curves show the trajectories of individual feature coefficients as the penalty parameter changes. Different colored lines represent different features. The bottom x-axis represents Log(λ), and the top x-axis indicates the number of features with non-zero coefficients. (B) Cross-validation curve. The curve shows the binomial deviance across different penalty parameter values. The bottom x-axis represents Log(λ), and the top x-axis indicates the number of features with non-zero coefficients. Two dashed vertical lines indicate the candidate optimal penalty parameters: the left line corresponds to λ.min, which gives the minimum binomial deviance, and the right line corresponds to λ.1se, which represents the most parsimonious model within 1 standard error of the minimum.
